# Sacituzumab govitecan and radiotherapy in metastatic, triple-negative, and BRCA-mutant breast cancer patient with active brain metastases: A case report

**DOI:** 10.3389/fonc.2023.1139372

**Published:** 2023-02-20

**Authors:** Pierluigi di Mauro, Greta Schivardi, Rebecca Pedersini, Lara Laini, Andrea Esposito, Vito Amoroso, Marta Laganà, Salvatore Grisanti, Deborah Cosentini, Alfredo Berruti

**Affiliations:** ^1^ Medical Oncology, Azienda Socio Sanitaria Territoriale Spedali Civili, Brescia, Italy; ^2^ Breast Unit, Azienda Socio Sanitaria Territoriale Spedali Civili, Brescia, Italy

**Keywords:** sacituzumab govitecan, triple-negative breast cancer, brain metastases, BRCA2, antibody-drug conjugate

## Abstract

**Background:**

Triple-negative breast cancer (TNBC) is an aggressive cancer subtype, owing to its high metastatic potential: Patients who develop brain metastases (BMs) have a poor prognosis due to the lack of effective systemic treatments. Surgery and radiation therapy are valid options, while pharmacotherapy still relies on systemic chemotherapy, which has limited efficacy. Among the new treatment strategies available, the antibody-drug conjugate (ADC) sacituzumab govitecan has shown an encouraging activity in metastatic TNBC, even in the presence of BMs.

**Case presentation:**

A 59-year-old woman was diagnosed with early TNBC and underwent surgery and subsequent adjuvant chemotherapy. A germline pathogenic variant in BReast CAncer gene 2 (BRCA2) was revealed after genetic testing. After 11 months from the completion of adjuvant treatment, she had pulmonary and hilar nodal relapse and began first-line chemotherapy with carboplatin and paclitaxel. However, after only 3 months from starting the treatment, she experienced relevant disease progression, due to the appearance of numerous and symptomatic BMs. Sacituzumab govitecan (10 mg/kg) was started as second-line treatment as part of the Expanded Access Program (EAP). She reported symptomatic relief after the first cycle and received whole-brain radiotherapy (WBRT) concomitantly to sacituzumab govitecan treatment. The subsequent CT scan showed an extracranial partial response and a near-to-complete intracranial response; no grade 3 adverse events were reported, even if sacituzumab govitecan was reduced to 7.5 mg/kg due to persistent G2 asthenia. After 10 months from starting sacituzumab govitecan, a systemic disease progression was documented, while intracranial response was maintained.

**Conclusions:**

This case report supports the potential efficacy and safety of sacituzumab govitecan in the treatment of early recurrent and BRCA-mutant TNBC. Despite the presence of active BMs, our patient had a progression-free survival (PFS) of 10 months in the second-line setting and sacituzumab govitecan was safe when administered together with radiation therapy. Further real-world data are warranted to confirm sacituzumab govitecan efficacy in this patient population.

## Introduction

Triple-negative breast cancer (TNBC), characterized by the lack of expression of the estrogen receptor (ER), progesterone receptor (PgR), and human epidermal growth factor receptor 2 (HER2), accounts for approximately 12–15% of breast cancers diagnosed worldwide (1–3). Despite extensive studies that have led to a better understanding of its clinical and biological heterogeneity (4–6), TNBC remains the most aggressive breast cancer subtype, owing to its high visceral metastatic potential, especially to the lungs and brain (7): The median overall survival (OS) is 10–13 months in the metastatic setting (1).

A recent meta-analysis highlighted that approximately one-third of patients with metastatic TNBC will eventually develop brain metastases (BMs) (8). The main current therapeutic options for BMs in TNBC are surgery and radiation therapy, either stereotactic radiosurgery (SRS) or whole-brain radiotherapy (WBRT) (9, 10): In particular, WBRT should be the favored choice for multiple BMs not amenable to SRS, due to neurological symptoms, size, number, and/or location (10).

BM pharmacotherapy of patients with TNBC remains challenging due to the lack of targeted therapies and the difficulties associated with drug delivery to the brain. Moreover, few data are available on the role of systemic treatments because patients with BMs have been generally excluded from clinical trials for several reasons, such as limited penetration of agents through the blood–brain barrier, difficulties in monitoring the response, and typically poor prognosis (11). Cytotoxic chemotherapy remains the mainstay of systemic treatment for BMs in TNBC, with an objective response rate (ORR) of about 30% (9, 10, 12), and different chemotherapy agents have been employed, such as taxanes, anthracyclines, etoposide, platinum compounds, capecitabine, and temozolomide (12–14).

For this reason, various efforts have been made to develop new therapeutic options and to identify molecular biomarkers, with the purpose of improving the clinical outcomes of patients with TNBC (15). From the expression of the programmed death-ligand 1 (PD-L1), patients who are more likely to benefit from the association between an immune checkpoint inhibitor (ICI) and chemotherapy in the metastatic setting may be selected (16, 17). However, the benefit for patients with BMs is uncertain.

Additionally, nearly 15% of patients with TNBC harbor a germline mutation of BRCA1 and/or BRCA2 (18). Although these patients may receive benefit from poly(ADP-ribose) polymerase (PARP) inhibitors, such as olaparib and talazoparib (19, 20), no data are available on the efficacy of PARP inhibitors in TNBC patients with BMs and new agents able to cross the blood–brain barrier are under development (21).

Sacituzumab govitecan is a first-in-class antibody-drug conjugate (ADC) that targets the human trophoblast cell-surface antigen (Trop-2), which is expressed on approximately 90% of TNBCs, and delivers its payload based on SN-38, the active metabolite of irinotecan (22, 23). The phase III ASCENT trial demonstrated a significant improvement over standard chemotherapy with respect to median progression-free survival (PFS) and median OS in TNBC patients who had received at least two chemotherapy regimens for advanced disease (24). TNBC patients with stable BMs were eligible to enter the trial, but they were a small cohort (61 patients) and excluded from the primary analysis; patients with active BMs were not eligible. Furthermore, in this trial, only 7% of patients had BRCA1/2 mutations and information on BRCA status was lacking in 38% of study population (24).

In this report, we present the clinical course and outcomes of a metastatic, early recurrent, TNBC patient, with a BRCA2 mutation and active BMs, who showed a remarkable response to radiotherapy combined with sacituzumab govitecan as second-line treatment.

## Case presentation

In December 2019, a 59-year-old woman presented with a left breast mass measuring 13 mm. Twenty-seven years before presentation, when she was 32 years old, she was diagnosed with stage II triple-negative left breast cancer and was treated with surgery, adjuvant chemotherapy, and radiotherapy; after 12 years (15 years before presentation), she was diagnosed with contralateral stage II TNBC and was treated similarly with surgery, adjuvant anthracycline-based chemotherapy, and radiotherapy. Her family history was notable for breast cancer in her maternal aunt.

An ultrasound-guided core biopsy showed grade 3, invasive TNBC, and a Ki-67 expression of 90%. Preoperative staging with CT scan did not show other metastatic lesions. She underwent a left-sided skin-sparing mastectomy and subsequently completed adjuvant chemotherapy with epirubicin and cyclophosphamide followed by weekly paclitaxel. A germline genetic testing was performed, which revealed the presence of a pathogenic variant in BRCA2 (8765delAG); however, no adjuvant PARP inhibitor therapy was available at that time. Due to the BRCA2 germline pathogenic variant, she underwent risk-reducing bilateral salpingo-oophorectomy 10.5 months after completing adjuvant treatment. A routinary chest CT scan after surgery revealed the appearance of two right pulmonary nodules (5 and 9 mm) and a subsequent 18F-FDG PET confirmed their high metabolic activity and demonstrated pathological uptake in the right hilar lymph nodes. A bronchoscopy with fine-needle aspiration cytology of the lymph nodes assessed the presence of neoplastic cells, whose morphology was attributed to breast carcinoma. Immunohistochemical studies confirmed TNBC and the PD-L1 expression (Ventana SP142) was < 1%. Brain CT scan was negative. The disease-free survival (DFS) in the adjuvant setting was 18.5 months.

With her score being ‘0’ on the Eastern Cooperative Oncology Group performance status (ECOG PS) scale, in August 2021, the patient began first-line chemotherapy with carboplatin AUC 2 and paclitaxel at 90 mg/m^2^ on days 1, 8, 15 every 28 days. After 3 months from starting chemotherapy, the patient reported a progressive onset of headache: Brain CT scan showed the appearance of numerous lesions, both in the cerebellum (10-mm diameter in the right hemisphere) and in the supratentorial region (5 mm in the parietal and frontal lobes, bilaterally). The radiological evaluation also documented pulmonary, hilar nodal, bone, and bilateral adrenal disease progression, resulting in a PFS of 3.5 months after the first-line treatment.

Her ECOG PS score then became ‘1’ as she did not complain further symptoms, apart from the headache. Considering the unavailability of clinical trials in our hospital at that time and the patient’s preference to continue systemic therapy, sacituzumab govitecan was requested as part of the Expanded Access Program (EAP). Treatment was approved by the local ethics committee and the patient provided written informed consent prior to the initiation of treatment.

Sacituzumab govitecan, at 10 mg/kg (on days 1 and 8 every 21 days), was started as second-line treatment in January 2022. After the completion of the first cycle, the patient described a rapid clinical benefit and reported a reduction both of the headache intensity and of the need for corticosteroids. Nonetheless, due to the extensive central nervous system (CNS) involvement and the uncertainty with respect to the depth and the duration of intracranial clinical response, the patient also received WBRT (30 Gy in 10 fractions), starting 2 days after day 8 of the first cycle. Treatment with sacituzumab govitecan was restarted 8 days after the end of WBRT upon patient’s request. The CT scan restaging after three cycles of sacituzumab govitecan showed a significant partial response on all disease sites and a near-to-complete intracranial response. Considering the absence of new lesions and/or edema after WBRT, treatment with corticosteroids was gradually tapered and stopped 21 days after the end of radiotherapy. Her PS remained good and treatment tolerance was globally acceptable, with the prevalent side effects of grade 1 (G1) or 2 (G2): G2 asthenia, G1 diarrhea, G2 neutropenia, and G1 dry skin. However, after the completion of four cycles of treatment, G2 asthenia persisted despite supportive treatment (reintroduction of low-dose corticosteroids and ginseng supplements) and made a significant impact on the patient’s quality of life; therefore, in agreement with the patient, sacituzumab govitecan was reduced to 7.5 mg/kg and was continued at this dose. No further relevant adverse events emerged during treatment; after 10 months from starting sacituzumab govitecan, a CT scan documented systemic disease progression, while intracranial response was maintained.

**Figure 1:**
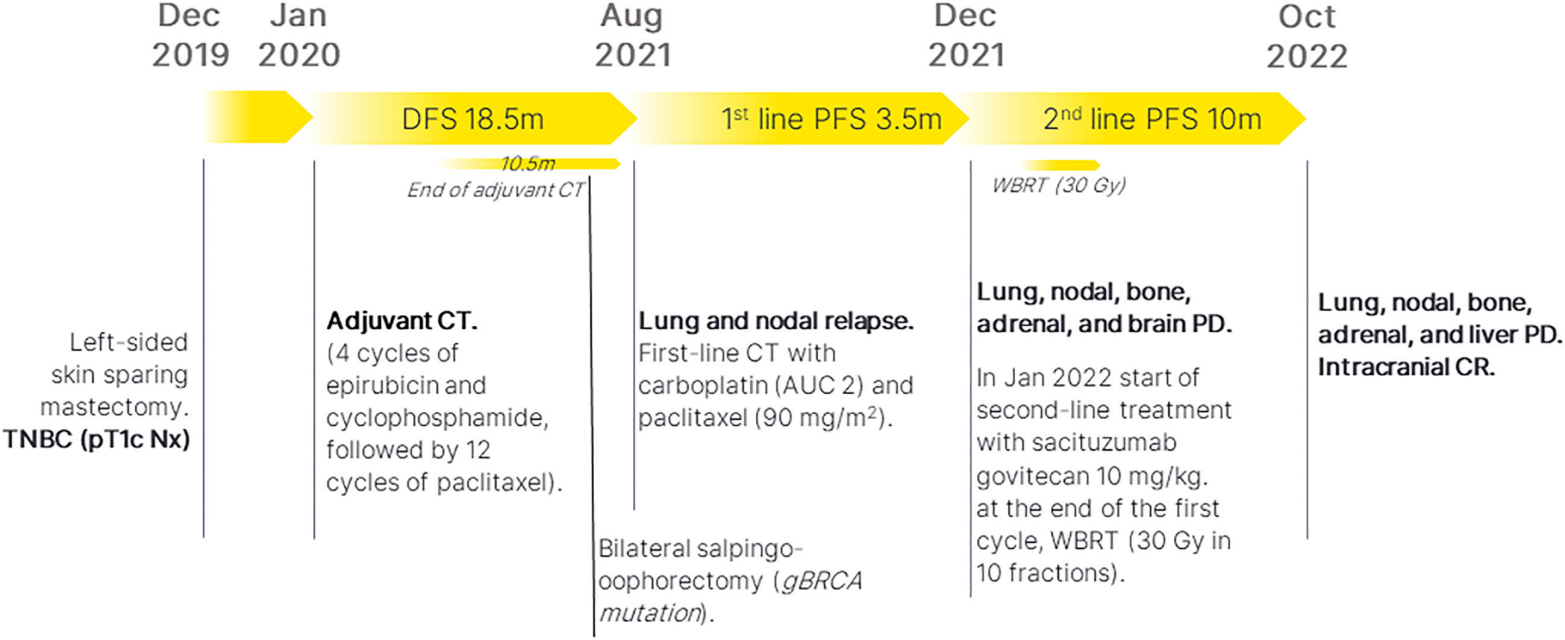
Patient’s timeline. TNBC, triple-negative breast cancer; CT, chemotherapy; DFS, disease-free survival; PFS, progression-free survival; PD, progressive disease; CR, complete response; WBRT, whole-brain radiotherapy; m, months.

**Figure 2:**
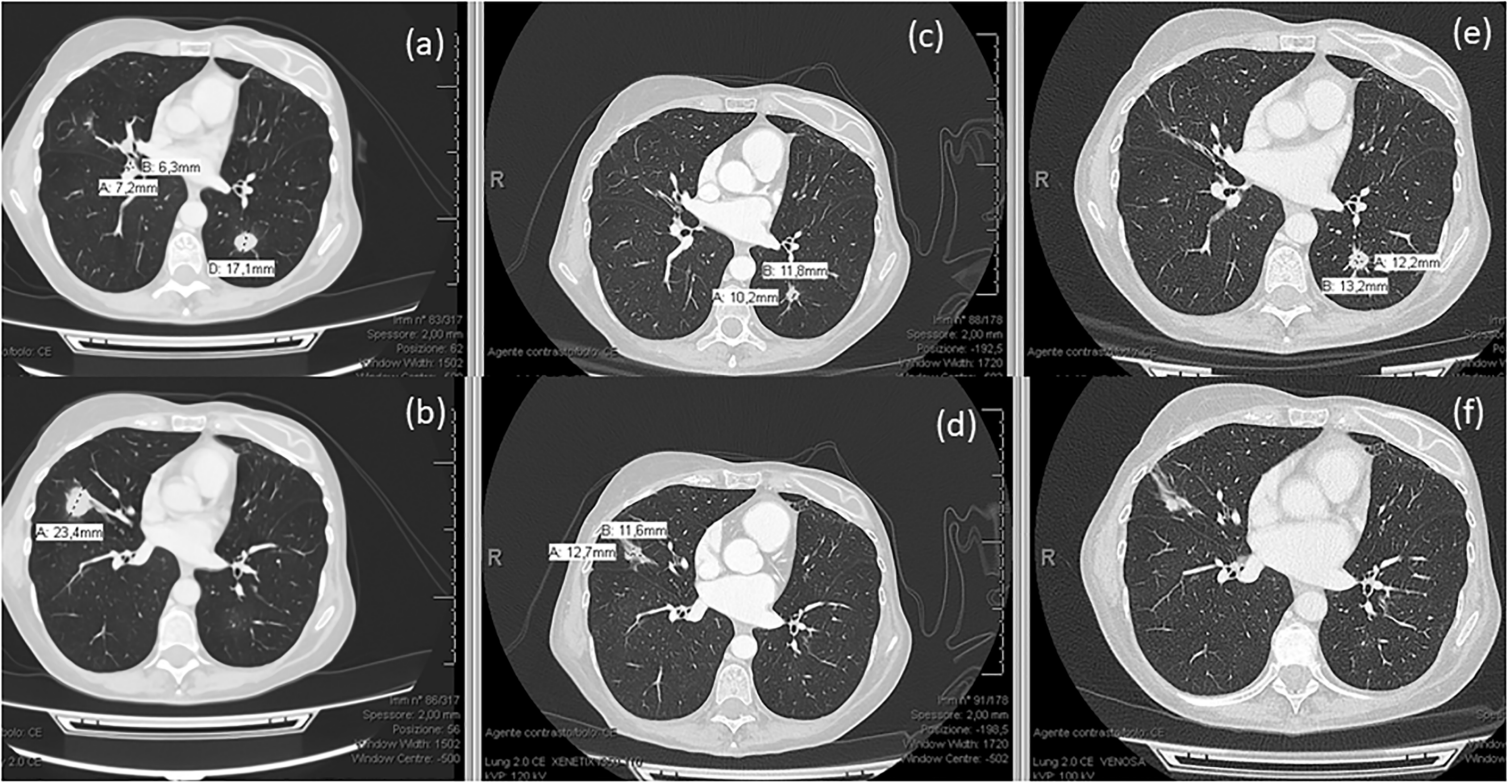
Radiological evaluation of the patient’s lung metastases **(A, B)** before, **(C, D)** after 3.5 months, and **(E, F)** after 7 months of treatment with sacituzumab govitecan.

**Figure 3:**
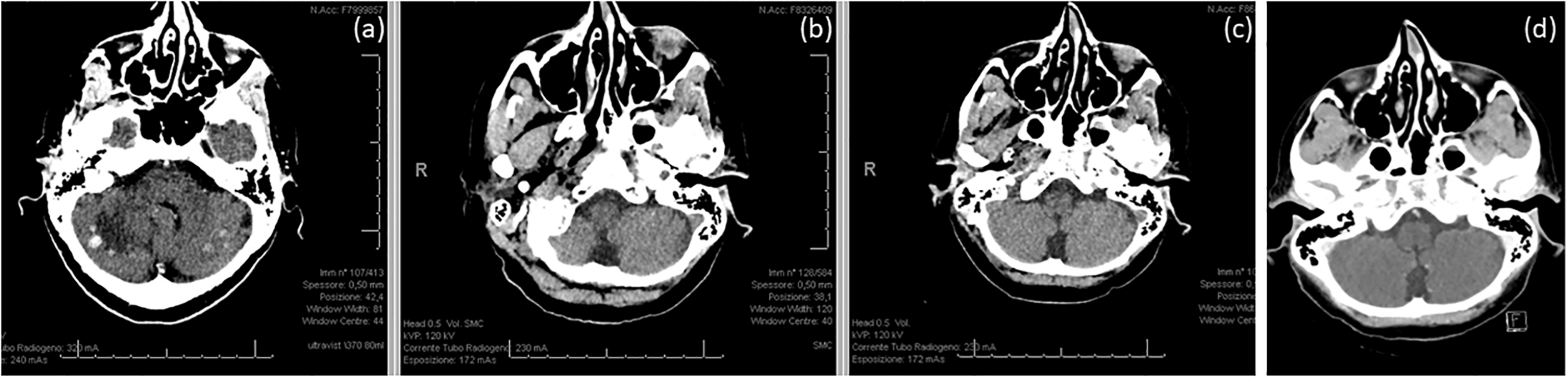
Intracranial disease **(A)** before, **(B)** after 3.5 months, and **(C)** after 7 months of treatment with sacituzumab govitecan. **(D)** Intracranial response was maintained after systemic progression.

Our patient’s timeline is reported in **Figure 1** and the radiological evaluations of extracranial (lung) and intracranial response during sacituzumab govitecan treatment are reported in **Figures 2**, **3**.

## Discussion

This case report outlines the efficacy of sacituzumab govitecan as a second-line treatment in a patient with metastatic TNBC, who harbors a BRCA2 mutation and active BMs.

Patients with BRCA-mutant TNBC have an increased susceptibility to DNA-damaging drugs, such as platinum compounds (25); indeed, the TNT trial demonstrated a double ORR with carboplatin *versus* docetaxel in subjects with BRCA-mutant metastatic TNBC (68% *vs.* 33%, respectively) (26).

However, the European Society for Medical Oncology (ESMO) guidelines recommend a PARP inhibitor as first-line treatment in this patient population (12): In fact, subjects who received first-line olaparib had a greater OS benefit compared with standard chemotherapy in the OlympiAD trial (27) and PARP inhibitor therapy has confirmed its broad efficacy in a recent meta-analysis, either as a single agent or combined with other drug classes (28). Nevertheless, in Italy, treatment with PARP inhibitor for metastatic breast cancer is allowed only after failure of platinum-based chemotherapy; therefore, our patient who relapsed after 11 months from adjuvant anthracycline- and taxane-based chemotherapy, started first-line treatment with carboplatin and paclitaxel.

Our patient developed rapid, symptomatic, and diffuse BMs after only 3 months from starting chemotherapy. It is well known that one-third of patients with metastatic TNBC will eventually develop BMs (8): As opposed to the HER2-positive breast cancer counterpart, for which several target therapies exist (29), drugs with potential intracranial efficacy are not available for TNBC and are under investigation (9, 15).

Our patient experienced a quick disease progression and had a high brain tumor burden: Since platinum-refractory diseases were excluded from the main PARP inhibitor clinical trials and their intracranial activity is uncertain, we preferred to start sacituzumab govitecan as a second-line treatment in the EAP. The phase III ASCENT trial enrolled TNBC patients who had received two or more lines of chemotherapy in the metastatic setting (24). Patients were randomized to receive sacituzumab govitecan *versus* chemotherapy of the physician’s choice (eribulin, vinorelbine, capecitabine, or gemcitabine): The control arm did not employ platinum-based compounds and only a minority of patients were BRCA-mutant (7%). Even if patients with BMs at baseline were accepted, the primary endpoint analysis did not include this patient population. The study showed a significant benefit of sacituzumab govitecan *versus* chemotherapy with respect to the median PFS (5.7 *vs.* 1.7 months; hazard ratio, (HR) 0.41; *p* < 0.001) and median OS (12.1 *vs.* 6.7 months; HR, 0.48; *p* < 0.001) (24).

An exploratory sub-analysis of the ASCENT study assessed the efficacy of sacituzumab govitecan as second-line treatment, namely, patients who received one line of therapy in the metastatic setting and recurred ≤ 12 months after (neo)adjuvant chemotherapy prior to study enrollment. The benefit in PFS and OS was consistent with the results of the ASCENT trial (30). Our patient, who could be part of this cohort, experienced an excellent PFS of 10 months, despite having active BMs at the start of the treatment.

Moreover, a recent network meta-analysis showed the superiority of sacituzumab govitecan on all endpoints compared with other treatments for TNBC in second/further lines (31). Taken together, these data strongly support sacituzumab govitecan being the preferred second-line treatment in metastatic TNBC.

Regarding safety, the most common grade 3 (G3) adverse events in the pivotal trial for sacituzumab govitecan were neutropenia (63%), diarrhea (59%), nausea (57%), alopecia (46%), and asthenia/fatigue (45%) (24). Our patient experienced good treatment tolerance, reporting only G1–G2 treatment–related adverse events; of note, no hematological or gastrointestinal G3 adverse events occurred, but persistent G2 asthenia was the most relevant, due to which sacituzumab govitecan was reduced to 7.5 mg/kg after four cycles.

It is not clear if these symptoms were entirely treatment related or caused by the association between sacituzumab govitecan and WBRT: In fact, our patient continued to receive sacituzumab govitecan and no data exist regarding the safety of this concomitant approach.

However, we hypothesize that this treatment combination allowed our patient to achieve a clinically relevant symptomatic relief and a near-to-complete response on BMs as evidenced by the preliminary data on CNS penetration of sacituzumab govitecan (32) and the enhanced drug concentrations in brain parenchyma after WBRT. BMs from breast cancer remain a therapeutic challenge and new medical strategies are currently under investigation (9, 11): Among these, ADCs have shown encouraging results, even when administered concomitantly with radiotherapy in the context of HER2-positive disease (33). However, medical therapy of BMs specifically from TNBC is lacking in new strategies, as only data from small studies with the addition of bevacizumab to chemotherapy have been reported (34–36). For these reasons, the administration of the ADC sacituzumab govitecan in patients with BMs from TNBC may be worth further investigation. Although intracranial response was not a dedicated endpoint in the ASCENT trial, an exploratory analysis including patients with stable BMs at screening showed a numerically better ORR and PFS for sacituzumab govitecan, but not OS (37).

Finally, Trop-2 expression by immunohistochemistry was not available: Despite patients with high or medium Trop-2 expression having had more favorable outcomes, a recent biomarker analysis of the ASCENT trial suggested that this feature may not be needed to predict patient response (38). Notably, the same analysis emphasized the efficacy of sacituzumab govitecan regardless of germline BRCA mutation status (38).

To summarize, our patient experienced a PFS of 10 months after radiotherapy and sacituzumab govitecan as second-line treatment, which was better compared with the median PFS from the pivotal trial (5.7 months), despite the presence of active BMs. The best overall response was extracranial partial response and near-to-complete intracranial response. She is now a candidate to start a new line of therapy, either with a PARP inhibitor or with a different chemotherapy regimen.

## Conclusions

The present case report supports a potential role for sacituzumab govitecan in the treatment of early recurrent and BRCA-mutant TNBC. Moreover, sacituzumab govitecan showed a high activity in active BMs and was globally safe when administered concomitantly with WBRT in our patient. So far, no experiences about radiotherapy and concurrent sacituzumab govitecan are described.

This evidence suggests its indication and use in the early steps of the systemic treatment sequence: However, real-world data are warranted to confirm its efficacy and safety in metastatic TNBC when administered with radiotherapy, either as SRS or WBRT.

## Data availability statement

The original contributions presented in the study are included in the article, further inquiries can be directed to the corresponding author (p.dimauro001@unibs.it).

## Ethics statement

The studies involving human participants were reviewed and approved by Comitato Etico Spedali Civili di Brescia. The patients/participants provided their written informed consent to participate in this study.

## Author contributions

PdM, GS, and RP designed the case report and wrote the first draft of the manuscript. LL, AE, and ML gathered the clinical and radiological data. VA, SG, DC, and AB contributed to the critical revision of the manuscript. All authors contributed to the article and approved the submitted version.
